# Author Correction: Repurposing type I–F CRISPR–Cas system as a transcriptional activation tool in human cells

**DOI:** 10.1038/s41467-020-17379-y

**Published:** 2020-07-09

**Authors:** Yuxi Chen, Jiaqi Liu, Shengyao Zhi, Qi Zheng, Wenbin Ma, Junjiu Huang, Yizhi Liu, Dan Liu, Puping Liang, Zhou Songyang

**Affiliations:** 1 Sun Yat-Sen Memorial Hospital, Sun Yat-sen University; MOE Key Laboratory of Gene Function and Regulation and Guangzhou Key Laboratory of Healthy Aging Research, School of Life Sciences, Sun Yat-sen University, Guangzhou, 510275 China; 2 State Key Laboratory of Ophthalmology, Zhongshan Ophthalmic Center, Sun Yat-sen University, Guangzhou, 510060 China; 3 Key Laboratory of Reproductive Medicine of Guangdong Province, the First Affiliated Hospital and School of Life Sciences, Sun Yat-sen University, Guangzhou, 510275 China; 4 Verna and Marrs Mclean Department of Biochemistry and Molecular Biology, Baylor College of Medicine, One Baylor Plaza, Houston, TX 77030 USA; 5 Guangzhou Regenerative Medicine and Health-Guangdong Laboratory (GRMH-GDL), Guangzhou, 510530 China

**Keywords:** Biochemistry, CRISPR-Cas systems, Biological techniques, Molecular engineering

Correction to: *Nature Communications* 10.1038/s41467-020-16880-8; published online 19 June 2020.

The original version of this Article contained an error in the author affiliations.

Affiliation 1 incorrectly read ‘The Second Affiliated Hospital, Sun Yat-sen University; MOE Key Laboratory of Gene Function and Regulation and Guangzhou Key Laboratory of Healthy Aging Research, School of Life Sciences, Sun Yat-sen University, Guangzhou, 510275, China’ instead of the correct ‘Sun Yat-Sen Memorial Hospital, Sun Yat-sen University; MOE Key Laboratory of Gene Function and Regulation and Guangzhou Key Laboratory of Healthy Aging Research, School of Life Sciences, Sun Yat-sen University, Guangzhou, 510275, China’.

This has now been corrected in both the PDF and HTML versions of the Article.

