# Supplementary material for: Repurposing type I–F CRISPR–Cas system as a transcriptional activation tool in human cells
Source: Nat Commun. 2020 Jun 19;11:3136. doi: 10.1038/s41467-020-16880-8 (PMC7305327; doi:10.1038/s41467-020-16880-8)
Supplement: Supplementary file 2 — Reporting Summary [file 41467_2020_16880_MOESM2_ESM.pdf]

nature research

Corresponding author(s): Zhou Songyang

Last updated by author(s): May 6, 2020

# Reporting Summary

Nature Research wishes to improve the reproducibility of the work that we publish. This form provides structure for consistency and transparency in reporting. For further information on Nature Research policies, see our Editorial Policies and the Editorial Policy Checklist.

## Statistics

For all statistical analyses, confirm that the following items are present in the figure legend, table legend, main text, or Methods section.

| n/a | Confirmed | |
|---|---|---|
| ☐ | ☒ | The exact sample size ($n$) for each experimental group/condition, given as a discrete number and unit of measurement |
| ☐ | ☒ | A statement on whether measurements were taken from distinct samples or whether the same sample was measured repeatedly |
| ☐ | ☒ | The statistical test(s) used AND whether they are one- or two-sided<br>*Only common tests should be described solely by name; describe more complex techniques in the Methods section.* |
| ☒ | ☐ | A description of all covariates tested |
| ☒ | ☐ | A description of any assumptions or corrections, such as tests of normality and adjustment for multiple comparisons |
| ☐ | ☒ | A full description of the statistical parameters including central tendency (e.g. means) or other basic estimates (e.g. regression coefficient) AND variation (e.g. standard deviation) or associated estimates of uncertainty (e.g. confidence intervals) |
| ☐ | ☒ | For null hypothesis testing, the test statistic (e.g. $F$, $t$, $r$) with confidence intervals, effect sizes, degrees of freedom and $P$ value noted<br>*Give P values as exact values whenever suitable.* |
| ☒ | ☐ | For Bayesian analysis, information on the choice of priors and Markov chain Monte Carlo settings |
| ☒ | ☐ | For hierarchical and complex designs, identification of the appropriate level for tests and full reporting of outcomes |
| ☒ | ☐ | Estimates of effect sizes (e.g. Cohen's $d$, Pearson's $r$), indicating how they were calculated |

*Our web collection on statistics for biologists contains articles on many of the points above.*

## Software and code

Policy information about availability of computer code

| | |
|---|---|
| Data collection | no software was used for data collection |
| Data analysis | Data in all experiments were tested by GraphPad 7.0, and statistical significance was determined using the one-way ANOVA or Student t-test. |

For manuscripts utilizing custom algorithms or software that are central to the research but not yet described in published literature, software must be made available to editors and reviewers. We strongly encourage code deposition in a community repository (e.g. GitHub). See the Nature Research guidelines for submitting code & software for further information.

## Data

Policy information about availability of data

All manuscripts must include a data availability statement. This statement should provide the following information, where applicable:

- Accession codes, unique identifiers, or web links for publicly available datasets
- A list of figures that have associated raw data
- A description of any restrictions on data availability

All relevant data are available upon request. Sequences of plasmids for expression of PaeCascade-VPR, including pCsy1-Csy2, pCsy3-VPR-Csy4, and pCsy-crRNA-EV, are listed in Supplementary Data 5. The source data for Figures. 1b, 1c, 1e, 1f, 1g, 2a, 2b, 2d, 3b, 3c, 3d, 3e, 4, 5b, 5c, 6b, 6d, 7 and Supplementary Figures. 2, 3b, 4b, and 7 are provided as a Source Data file.

# Field-specific reporting

Please select the one below that is the best fit for your research. If you are not sure, read the appropriate sections before making your selection.

- [x] Life sciences
- [ ] Behavioural & social sciences
- [ ] Ecological, evolutionary & environmental sciences

For a reference copy of the document with all sections, see nature.com/documents/nr-reporting-summary-flat.pdf

# Life sciences study design

All studies must disclose on these points even when the disclosure is negative.

| | |
|---|---|
| Sample size | The most experiments run in triplicate. The sample sizes were judged based on the reproducibility of measurements between groups..Statistical significance was determined using the one-way ANOVA or Student t-test. |
| Data exclusions | No data were excluded. |
| Replication | The most experiments were repeated three times. All attempts at replication were successful, and standard deviations were within expected ranges. |
| Randomization | Samples were randomly allocated into experimental groups. |
| Blinding | Investigators were not blinded during experiments and data analysis. |

# Reporting for specific materials, systems and methods

We require information from authors about some types of materials, experimental systems and methods used in many studies. Here, indicate whether each material, system or method listed is relevant to your study. If you are not sure if a list item applies to your research, read the appropriate section before selecting a response.

## Materials & experimental systems

| n/a | Involved in the study | |
|---|---|---|
| [ ] | [x] | Antibodies |
| [ ] | [x] | Eukaryotic cell lines |
| [x] | [ ] | Palaeontology and archaeology |
| [x] | [ ] | Animals and other organisms |
| [x] | [ ] | Human research participants |
| [x] | [ ] | Clinical data |
| [x] | [ ] | Dual use research of concern |

## Methods

| n/a | Involved in the study | |
|---|---|---|
| [x] | [ ] | ChIP-seq |
| [ ] | [x] | Flow cytometry |
| [x] | [ ] | MRI-based neuroimaging |

## Antibodies

| | |
|---|---|
| Antibodies used | Rabbit polyclonal anti-GAPDH (Abmart, P30008M) (1:5000 dilution), mouse monoclonal anti-HA antibody (sigma, H9658) (1:5,000 dilution), goat anti-rabbit secondary antibody (Odyssey, 926-32211) (1:5,000 dilution) and the goat anti-mouse secondary antibody (Odyssey, 926-68070) (1:5,000 dilution) |
| Validation | Rabbit polyclonal anti-GAPDH (Abmart, P30008M) (1:5000 dilution), suitable for WB indicated on the manufacturer's website;<br>Manufacturer's website: http://www.ab-mart.com.cn/page.aspx?node=59&id=994;<br>Also used in this study: TOE1 acts as a 3' exonuclease for telomerase RNA and regulates telomere maintenance.<br><br>mouse monoclonal anti-HA antibody (sigma, H9658), suitable for WB indicated on the manufacturer's website;<br>Manufacturer's website: https://www.sigmaaldrich.com/catalog/product/sigma/h9658?lang=en&region=US;<br>Also used in this study: pigenetic silencing of Oct4 by a complex containing SUV39H1 and Oct4 pseudogene lncRNA |

## Eukaryotic cell lines

Policy information about cell lines

| | |
|---|---|
| Cell line source(s) | HEK-293T cells was from ATCC. |
| Authentication | No eukarytoic cell lined were authenticated. |
| Mycoplasma contamination | All the cells have been tested negative for mycoplasma contamination by PCR. |

| | |
|---|---|
| Commonly misidentified lines (See ICLAC register) | No commonly misidentified cell lines were used. |

## Flow Cytometry

### Plots

Confirm that:

- [x] The axis labels state the marker and fluorochrome used (e.g. CD4-FITC).
- [x] The axis scales are clearly visible. Include numbers along axes only for bottom left plot of group (a 'group' is an analysis of identical markers).
- [x] All plots are contour plots with outliers or pseudocolor plots.
- [x] A numerical value for number of cells or percentage (with statistics) is provided.

### Methodology

| | |
|---|---|
| Sample preparation | Cultured HEK293T Cell were digested by 0.25 % trypsin, and then trypsin digestion was terminated by DMEM containing 10% FBS. Cells were collected and suspended in PBS for determination of GFP positive cells. |
| Instrument | CytoFLEX (Beckman) |
| Software | CytExpert 2.0 |
| Cell population abundance | Percentage of GFP positive cells in negative control was <1%, and the GFP positive populations ranged from <1% to 90% according to different treatments. GFP positive cells were confirmed under a florescent microscope. |
| Gating strategy | HEK293T cells were initially gated on population using FSC-A/SSC-A (Gate A) and then sorted for GFP positive cells using FITC-A/PE-A (Gate B). |

- [x] Tick this box to confirm that a figure exemplifying the gating strategy is provided in the Supplementary Information.
