# Supplementary material for: Repurposing type I–F CRISPR–Cas system as a transcriptional activation tool in human cells
Source: Nat Commun. 2020 Jun 19;11:3136. doi: 10.1038/s41467-020-16880-8 (PMC7305327; doi:10.1038/s41467-020-16880-8)
Supplement: Supplementary file 3 — Supplementary Information [file 41467_2020_16880_MOESM3_ESM.pdf]

**Supplementary Information**

# Repurposing Type I-F CRISPR-Cas System as a Transcriptional Activation Tool in Human Cells

Chen and Liu et al.

Supplementary Fig. 1

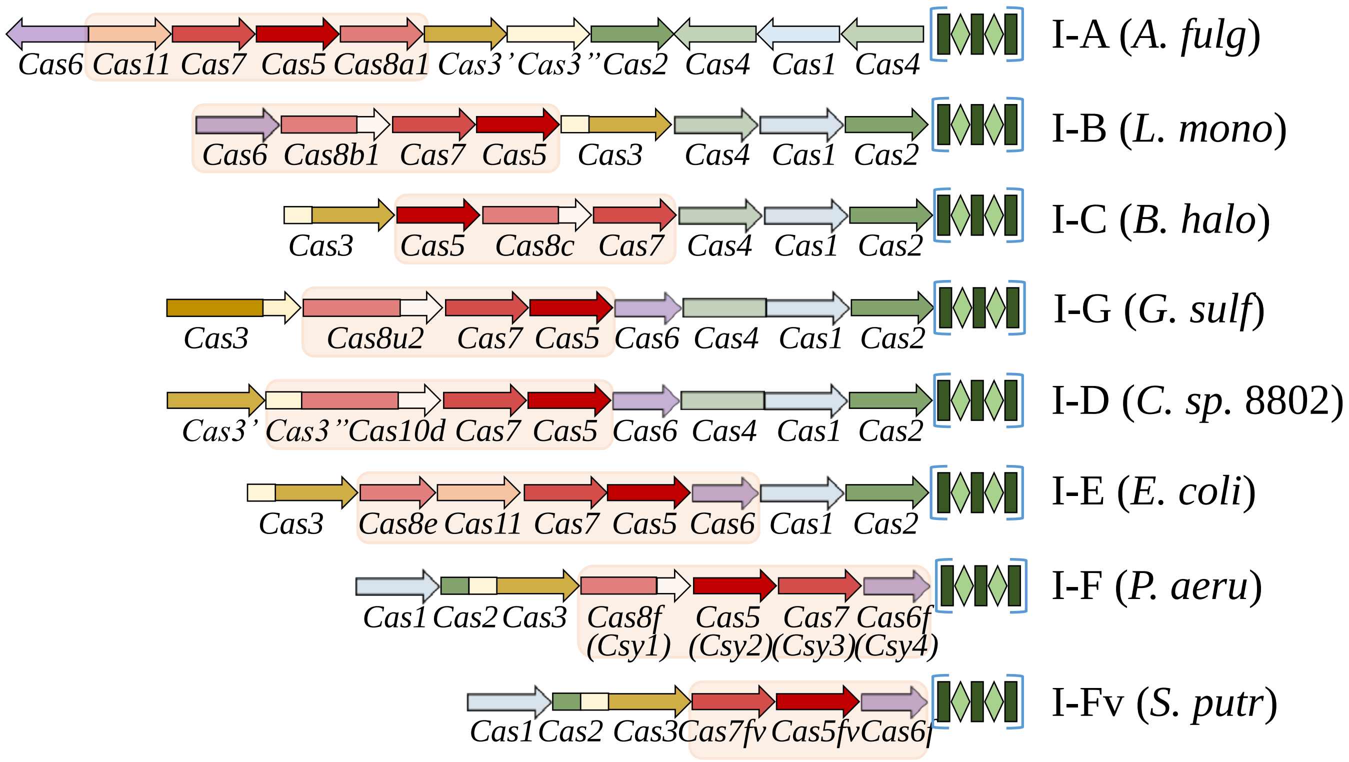


**Supplementary Figure 1. Updated classification of type I CRISPR-Cas system.**

Schematic of eight sub-types of class I type I CRISPR-Cas system. Arrows in different colors representing different Cas proteins. Genes for type I effector are shaded. The last array in green refers to CRISPR arrays. Type I-A (*Archaeoglobus fulgidus*: *A. fulg*) , Type I-B (*Listeria monocytogenes: L. mono*), Type I-C (*Bacillus halodurans*: *B. halo*), Type I-G (*Geobacter sulfurreducens*: *G. sulf*), Type I-D (*Cyanothece sp. 8802*: *C. sp.* 8802), Type I-E (*Escherichia coli K12*: *E. coli*), Type I-F (*Pseudomonas aeruginosaP. aeru*) , Type I-Fv (*Shewanella putrefaciens CN-32*: *S. putr*)

Supplementary Fig. 2

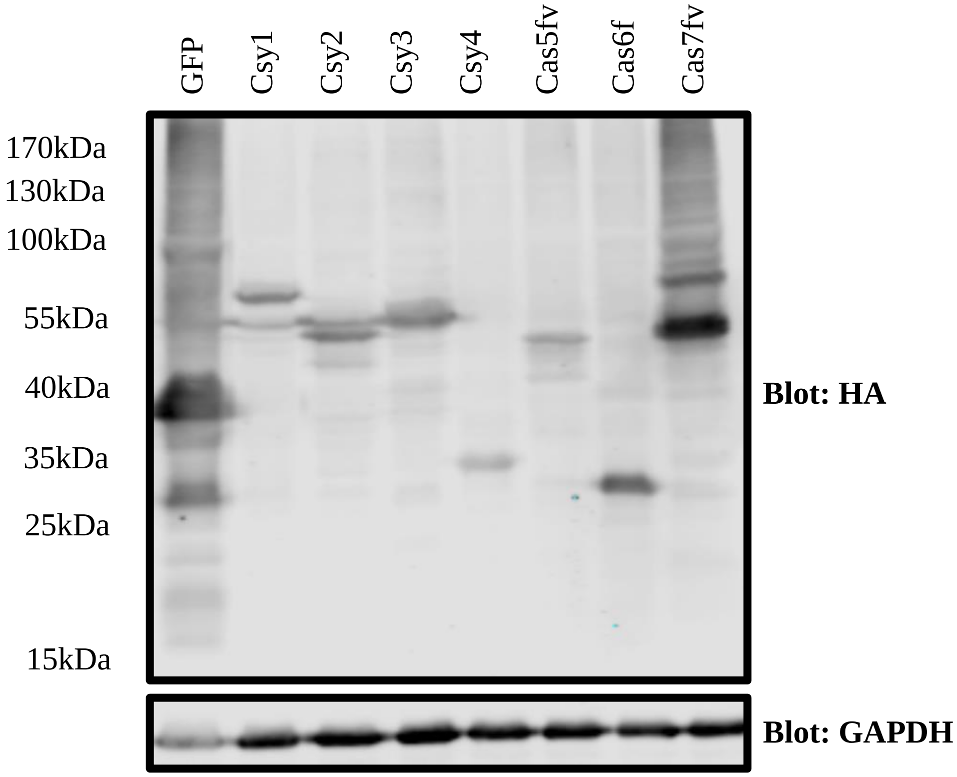


**Supplementary Figure 2. All type I-F and type I-Fv complex components could be expressed in the HEK293T.**

Western blot analysis of HEK293T cells transfected with individual plasmids containing HA-tagged type I-F or type I-Fv subunits. Cells were harvested 48 hours post-transfection. GFP served as control. Two independent experiments were conducted with similar results. Source data are provided as a Source Data file.

Supplementary Fig. 3

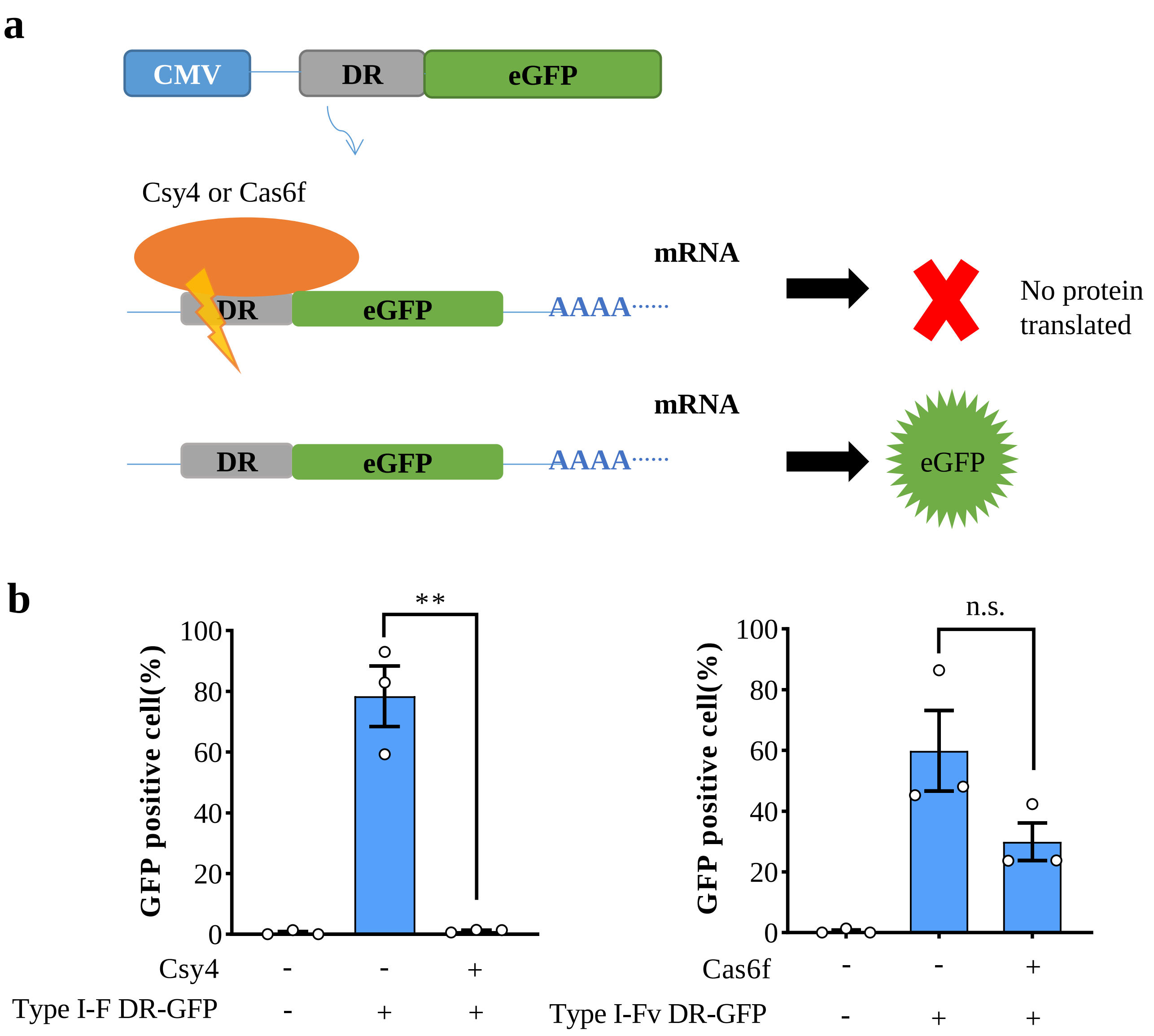

**Supplementary Figure 3. Csy4 and Cas6f could process pre-crRNA effectively in HEK293T.**

(**a**) Schematic illustrating the strategy for detecting pre-crRNA processing activity in HEK293T. The DRs (direct repeats) of Type I-F and Type I-Fv CRISPR were placed upstream GFP CDS driven by CMV promoter. When the DR-GFP mRNA, which would be translated into functional GFP, was transcripted, if Csy4 or Cas6f could recognize and cleave its DR sequences in HEK293T, the DR-GFP mRNA would be destroyed and no GFP signal could be detected.

(**b**) Flow cytometric analysis of GFP positive cells in HEK293T cotransfected with type I-F DR-GFP (or type I-Fv DR-GFP) and Csy4 (or Cas6fv). Vectors were 1:1 (Csy4(or Cas6fv):DR-GFP) in molar ratio for transfection in TRE-eGFP cells. Data was collected by flow cytometric analysis 48 hours post-transfection, and then total cells and GFP positive cells were counted. GFP positive cell ratio was determined by (GFP positive cell number / Total cell number). Ctrl: Untransfected control. Data represented three biological repeats and displayed as mean ± S.E.M. Statistical significance was calculated using one-way ANOVA . (n.s., not significant; **, $P < 0.01$). Source data are provided as a Source Data file.

Supplementary Fig. 4

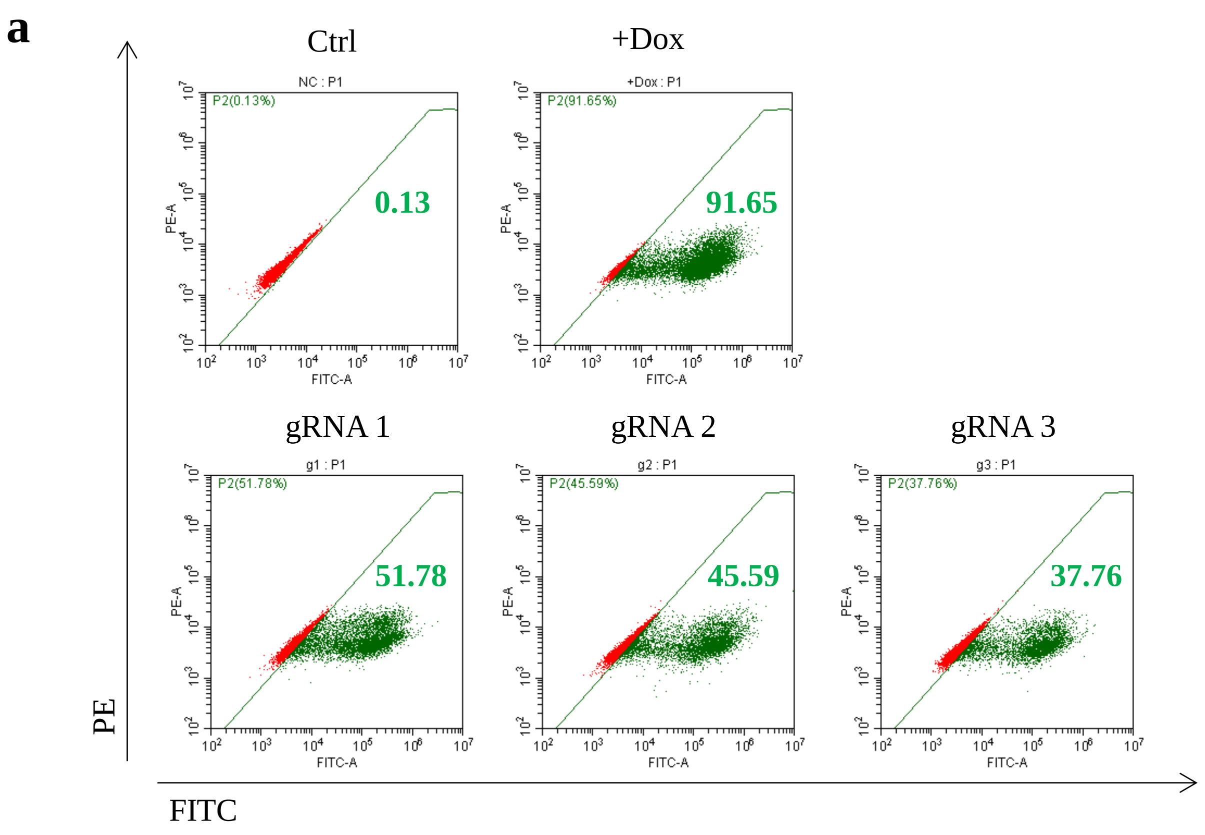


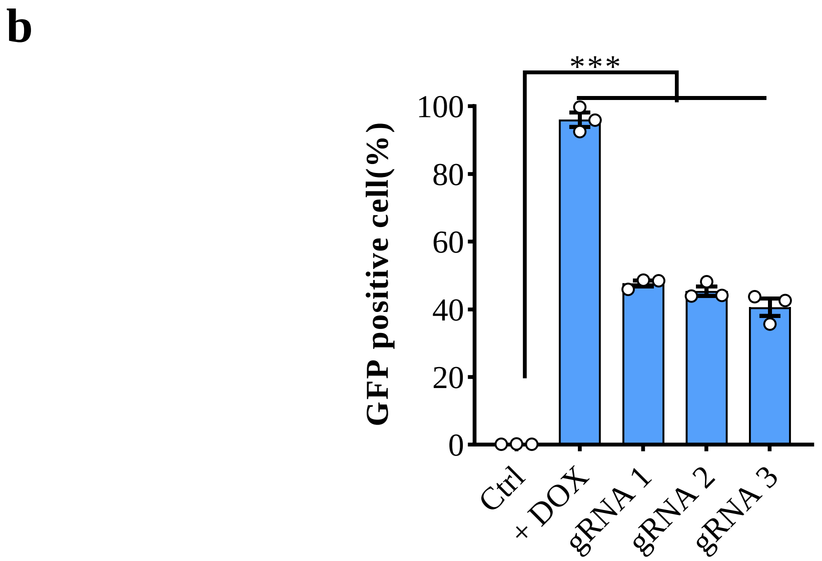

**Supplementary Figure 4. Inducible GFP expression in TRE-eGFP cells by dCas9-VPR.**

(**a**) Flow cytometric analysis of GFP activation in TRE-eGFP reporter cells induced by dox or transfected with dCas9-VPR/gRNA. TRE-eGFP reporter cell contains rtTA(reverse tetracycline-controlled trans activator). Data was collected in PE and FITC channel to eliminated auto-fluorescence. Percentages of GFP positive cells were labeled in green color.
(**b**) Quantification of GFP positive cell ratio induced by dCas9-VPR in TRE-eGFP reporter cells. Vectors were 1:1 (dCas9-VPR:gRNA) in molar ratio for transfection in TRE-eGFP cells. Data was collected by flow cytometric analysis 72 hours post-transfection, and then total cells and GFP positive cells were counted. GFP positive cell ratio was determined by (GFP positive cell number / Total cell number). Ctrl: Untransfected control. Data represented three biological repeats and displayed as mean ± S.E.M. Statistical significance was calculated using one-way ANOVA (***, $P < 0.001$). Source data are provided as a Source Data file.

Supplementary Fig. 5

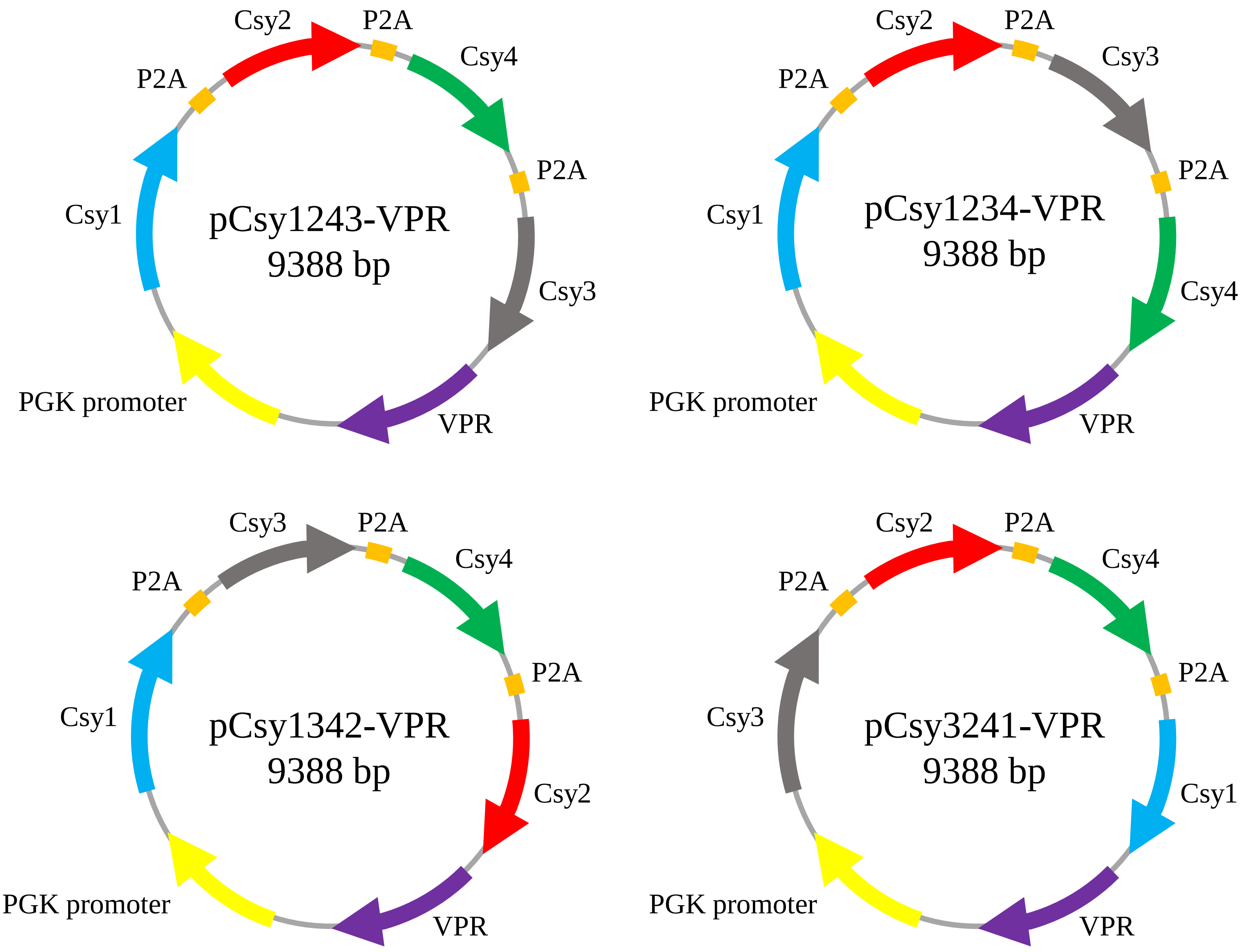


**Supplementary Figure 5. Map of the all-in-one type I-F system constructs.**

All the four Csy subunits were linked by P2A and driven by PGK promoter. The VPR transcriptional activator was fused with the last subunit. Selection marker and replication origin were not included in the maps.

Supplementary Fig. 6

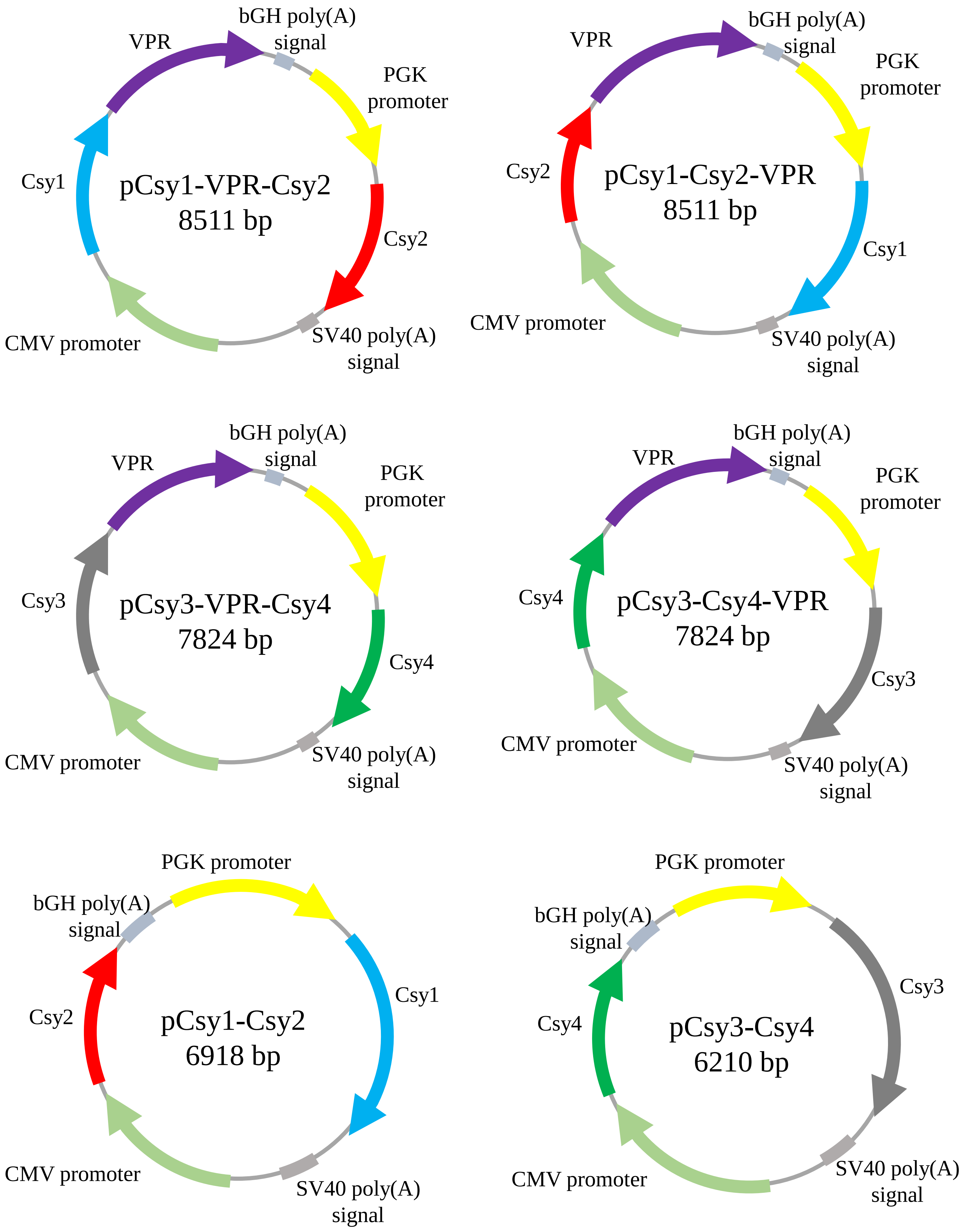

**Supplementary Figure 6. Map of type I-F system constructs with each Csy subunit driven by independent promoter**

Each construct includes two different subunits, and each subunit is driven by independent CMV promoter or PGK promoter. In pCsy1-VPR-Csy2, VPR is fused with Csy1. In pCsy1-Csy2-VPR, VPR is fused with Csy2. In pCsy3-VPR-Csy4, VPR is fused with Csy3. In pCsy3-Csy4-VPR, VPR is fused with Csy4. Selection marker and replication origin were not included in the maps.

Supplementary Fig. 7

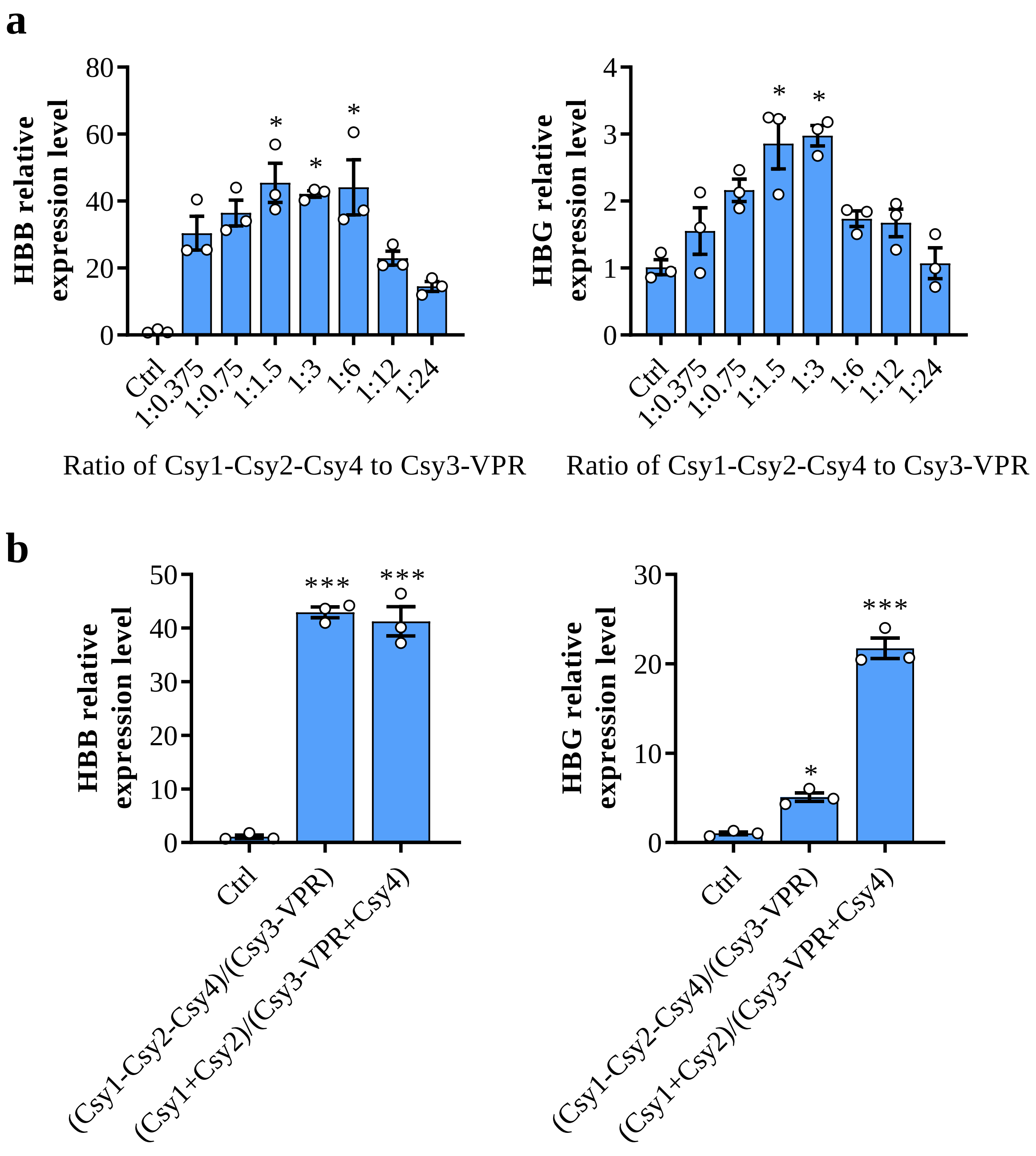

**Supplementary Figure 7. Activation of *HBB* or *HBG* using vectors with different arrangements of Csy subunits**

(**a**) Csy1, Csy2 and Csy4 were expressed by P2A fusion in one plasmid and Csy3 in another. The two plasmids were transfected with different molar ratios. (**b**) Comparison of the activation efficiency of two different constructs in activating *HBB* and *HBG*. Construct 1: Csy1, Csy2 and Csy4 were expressed by P2A fusion in one plasmid and Csy3 in another (molar ratio = 1:3). Construct 2: Csy1, Csy2 and Csy3-VPR, Csy4 were expressed in pairs on two plasmids (molar ratio = 1:1) with independent promoters. Data represented three biological repeats and displayed as mean ± S.E.M. Statistical significance was calculated using one-way ANOVA (*, $P < 0.05$; **, $P < 0.01$; ***, $P < 0.001$). Source data are provided as a Source Data file.
