# Supplementary material for: Repurposing type I–F CRISPR–Cas system as a transcriptional activation tool in human cells
Source: Nat Commun. 2020 Jun 19;11:3136. doi: 10.1038/s41467-020-16880-8 (PMC7305327; doi:10.1038/s41467-020-16880-8)
Supplement: Supplementary file 12 — Description of Additional Supplementary Files [file 41467_2020_16880_MOESM12_ESM.pdf]

Title: Supplementary Data 1
Description: crRNA sequences in Figure 1, 2, 4, 5, and 6

Title: Supplementary Data 2
Description: crRNA and gRNA sequences for activation efficiency comparison in Figure 3

Title: Supplementary Data 3
Description: the sequences of customized CRISPR arrays in Figure 5

Title: Supplementary Data 4
Description: crRNA and gRNA sequences for detecting off-target activations in Figure 7

Title: Supplementary Data 5
Description: complete sequences of pCsy1-Csy2, pCsy3-VPR-Csy4, and pCsy-crRNA-EV

Title: Supplementary Data 6
Description: DNA sequences for EMSA assay in Figure 1

Title: Supplementary Data 7
Description: quantitative PCR primers used in this study

Title: Supplementary Data 8
Description: predicted off-target sites in Figure 7
